# Communicating decisions about care with patients and companions in emergency department consultations

**DOI:** 10.1111/hex.13519

**Published:** 2022-06-17

**Authors:** Silvie Cooper, Fiona Stevenson

**Affiliations:** ^1^ Department of Applied Health Research University College London London UK; ^2^ Department of Primary Care and Population Health University College London London UK

**Keywords:** decision‐making, emergency department, junior doctors, patient communication, triadic communication

## Abstract

**Introduction:**

This paper explores doctor–patient and companion communication about care decisions in a UK emergency department (ED). Doctors interface between patients and healthcare systems and facilitate access to care across a range of encounters, drawing on information and authority to make and communicate clinical care decisions.

**Materials and Methods:**

We explored characteristics of communication through ethnographic observation of 16 video‐recorded case studies of ED consultations (average length: 1 h) collected over 6 months. Companions were present in 10 cases. We conducted a framework analysis to understand the roles of doctors, consultants, patients and companions in relaying ED care decisions.

**Findings:**

We present two cases to reflect companion roles and their effect on the consultation. The urgency for care and scarcity of resources means clinicians justify decisions and strategize to move patients along ED pathways.

**Discussion:**

Everyday care interactions between patients and doctors are goal‐oriented and companions participate by providing case information, querying decisions and advocating for care. Our findings reflect how doctors justify decisions made in communicating the next steps in ways that characterize the clinical encounter.

**Conclusion:**

By exploring everyday interactions our study contributes to a growing understanding of patient–clinician and companion communication in the ED.

**Patient or Public Contribution:**

Patients and caregivers voluntarily participated in data collection and consented to video recordings being conducted of ED consultations between them and junior doctors. There was extensive consultation with all grades of staff about the acceptability of the work and the best way to conduct it to minimize the impact on patients and staff. Through this manuscript, we have demonstrated the presence and important role of companions. On reflection it would have been valuable to have included patients and companions in discussions about the work; however, this project was conducted with very limited funding and no resources were committed to patient and public involvement. Given the setting and scope of the study, it was not feasible to involve patients or members of the public in other stages of the research or preparation of the manuscript. We recognize this as a potential limitation of the work.

## 1 INTRODUCTION

Unprecedented levels of demand for care in emergency departments (EDs) have increased the pressure on services to deliver safe, efficient and effective care while remaining mindful of hospital capacity and expectations around waiting times.^1^, ^2^, ^3^, ^4^ In this context, doctors justify choices about tests that are ordered (or not), referrals made to other departments for further care or discharge to community care as they navigate between patient needs and desires, and organizational priorities and practices. These justifications reflect a practice of medical gatekeeping, which can have facilitative or restrictive aspects and are ‘part of a coordinated organizational strategy for managing resource scarcity’.^5^ The need ‘for gatekeeping is usually underscored with three types of arguments: the need to ensure that patients receive *appropriate care*, the need for *budget restraints* and the need for *justice* in distributing care’^6^ (author's emphasis). ‘Justice’ here refers to the distribution of care based on those who are most in need.

Interactions between doctors and patients are purposeful and goal‐oriented, with the aim being to assess, treat and resolve the patient's case to move them through care pathways and out of ED.^7^, ^8^, ^9^ Communication plays a critical role in this process^5^, ^9^, ^10^, ^11^ and medical knowledge is constructed through a ‘joint project involving patients, professionals and society, and so involves a multiplicity of gazes’,^12^ which extends to medical practice.

In EDs, the use of medical investigations becomes an organizing principle—legitimizing a patient's presence in the space, validating or discounting diagnoses, affording access to care pathways and treatments and making health and illness visible and invisible, present and absent.^8^, ^13^, ^14^ Greaves^12^ critiques biomedicine and biopsychosocial approaches to health and illness for their sustained fragmentation and compartmentalization in organizing medical knowledge, patient experience and services. For Greaves^12^ this produces an underlying tension in integrating patients as holistic and complex people within specialized and differentiated practice and services towards common goals, arguing the result is that these tensions ‘resurface continually; however, much attention is paid to overcoming them’.

Different forms of information (patient‐reported symptoms and experience, physical examinations, medical investigations and the opinion of doctors, consultants and other clinical colleagues) are collated into a narrative and managed by the doctor.^8^, ^9^, ^12^, ^13^, ^15^, ^16^ The doctor needs to bring together and continually transform this narrative while accounting for wider system factors, such as capacity within the hospital and guidelines for accessing testing facilities.^1^, ^2^ While the ED is led by consultants and made up of multidisciplinary care teams of nurses, allied health professionals, care assistants, administrators and others, junior doctors are key facilitators of information flow. Junior doctors are ‘front‐line decision‐makers’^9^ in this context as they interface with and navigate between patients, consultants, clinical colleagues and wider institutional constraints in delivering care.^1^

Stevenson et al.^7^ argued for a nuanced understanding of what happens in EDs to identify the factors underlying patients' movement through this service. ‘Citing Goodwin^17^ Pelletier et al.^18^ noted that “medical practice is better represented as an ongoing flow to which multiple voices contribute, incurring dependencies on each other as contributions are interlaced; diagnoses and decision‐making are distributed, not independent, events’. These discursive practices also extend into doctor–patient and companion interactions and communication. Cox and Li^19^ explain that incorporating companions into the perspective of clinical communication sheds light on the ‘interactional and clinical reasoning aspects of medical consultations’. The role and influence of companions in medical consultations are largely underexplored, though companions are frequently present.^20^, ^21^, ^22^, ^23^, ^24^, ^25^

This paper extends the analyses of Stevenson et al.^7^ and Pelletier et al^18^ by focusing on case studies of consultations between junior doctors and patients and their companions to reflect the contribution of companions in shaping care decisions and communication in the ED.

## 2 MATERIALS AND METHODS

### 2.1 Study objectives and data collection

We aimed to understand communication in the ED between doctors, patients and their companions, and consultants, drawing on qualitative data from an ethnographic study conducted between 2014 and 2015 in an ED in southeast England. This study received ethical approval from a UK National Health Service (NHS) committee.^7^ The team conducted observations of doctors in the ED to understand junior doctors' decision‐making in this setting. Over 6 months, 16 case studies of consultations lasting an average of 1 h between doctors, patients and their companions (usually family) and consultants and other healthcare staff were collected through video recordings on static cameras located in the consultation room. A handheld camera operated by the researchers focused on the junior doctor outside the consultation room and a microphone worn by the junior doctor throughout was used to extend observation into general ED staff interactions.^7^

Care decisions are shaped by various factors, such as the complexity of the case presentation, readily available diagnoses and treatment plans, capacity within the health service and demand for care.^2^, ^4^, ^26^ We focused on the role of the junior doctor in directing the consultation with the patient and their companions and the communication mechanisms used to determine care pathways, and navigate and relate decisions across a range of people, organizational processes and contextual considerations.^11^ Our objective was to include cases that demonstrated a range of scenarios junior doctors encounter and address as frontline decision‐makers in this setting.^9^

The cases reflect examples of communication practices, care trajectories and pathways and roles seen across the data set. Companions were present in over half (*n* = 10) of the cases in the full data set. We focused our analysis on where doctors and patients and their family members interacted over multiple encounters to establish the reason for attending, convey key decisions and resolve care in ED.^7^

In the results, we present two exemplars of interaction between a patient, their family member, the doctor and a consultant in one case, and between a patient, their family member and the doctor in the other case. We demonstrate how companions influence communication with junior doctors by offering information about the patient, querying decisions made, and advocating for care.^25^

### 2.2 Analysis

We conducted a framework analysis, as the method facilitates the identification of patterns across cases and data and allows for the development of explanations for observations that are grouped around themes.^27^ We followed the stages described by Gale et al.^27^ to systematically familiarize ourselves with the data by reviewing all videos, audio recordings and transcripts of consultations between junior doctors, patients and their companions and consultants. Field notes of observations were gathered by watching the videos and reviewing transcripts. We then coded transcripts and fieldnotes to develop a working analytical framework in response to key questions related to *communicating decisions about care, and roles and practices of justifying resources and access to care pathways* and applied the framework to the data set. Our coding was cross‐referenced for an array of case presentations, decision‐making processes, care pathways and trajectories and communication between junior doctors, consultants, their patients and companions.^27^ Discussions between the study team facilitated the generation of codes, themes and analysis. One researcher coded all the data, with a second researcher checking the framework alongside, which allowed for the indexing, charting, mapping and interpretation of the data and findings.^27^ The framework is provided in Table A1.

## 3 FINDINGS

Across the study, doctors were focused on goal‐oriented objectives of assessing, offering treatment and care and explaining care decisions, and included companions in the consultation when needed. Companions were often quiet observers of the consultation. They could also usually offer information about the patient's symptoms and case history, prompt the patient to speak or act, or query the doctor's assessment and decisions about the patient's care. At times, patients deferred to their companions to speak on their behalf, and doctors frequently engaged with companions about different aspects of the patient's case and care.

### 3.1 Case 1: Younger man presenting with severe back pain

In this first case, a man had come to the ED with severe back pain, accompanied by his wife. The patient's wife listened intently while the doctor kept her attention focused on the patient to establish the nature of the patient's symptoms, and assessed the steps taken before arriving at the ED and medication received thus far. As the patient was explaining how much pain he was in and the doctor was about to commence a physical examination, his wife interjected:

**P's Wife:** I think he needs an MRI scan, you know, because … *[rubbing hand across forehead; **Junior doctor** looks over at wife]*.

**Patient:** I can't … I can't even twist.

**Jnr Doc:** OK. *[Turning attention and speaking to the patient's wife]* Why do you think he needs an MRI scan? Is there something in particular you're worried about?

**P's Wife:** Well, I nearly severed my spinal cord and that was completely misdiagnosed.

**Jnr Doc**: OK.

**P's Wife:** And it wasn't until the MRI scan that they realised that actually the disc had protruded and nearly cut off …

**Jnr Doc**: OK.

**P's Wife:** … the spinal cord. So, what I'm concerned about is that if there's anything that's […], you're not going to pick it up on the x‐ray, the only way you're going to get it is in an MRI.

**Jnr Doc**: OK.

**P's Wife:** And if he's having the sensation of pins and needles …! *[looks over at patient and makes a sweeping gesture with hand along direction down body, then turns it over, suggesting “if this, then that”]*.

**Jnr Doc:** Sure. Well, we'll do our clinical examination [on the patient] and then we'll decide whether or not one is necessary.

The patient's wife diverts the doctor into a discussion about accessing an magnetic resonance imaging (MRI) scan and what the most appropriate diagnostic route might be for the patient, given her misdiagnosis experience and the presence of pins and needles as a symptom for her husband. By relating her own experience and concerns, the patient's wife advocates for care for her husband, though the doctor is reluctant to engage in this investigative route and begins to act as a gatekeeper speaking about whether an MRI is ‘necessary’.^5^, ^25^ The doctor conducts the examination and explains that the priority is getting the patient's pain under control and that she will speak to the consultant about the patient's case. While outside the consultation room and away from the patient, the patient's wife approaches the junior doctor and says she must leave to collect her children and requests an update about her husband's care. The data presented below is drawn from the transcript of the audio recording of the junior doctor's microphone, so observation is not presented in this interaction. The junior doctor shares the next steps:

**Jnr Doc:** We're going to see if the medication we give now has any effect.

**P's Wife**: Mmn.

**Jnr Doc:** Erm, and then I'll talk to one of my seniors [the consultant]. But from what [the examination shows] … I don't think [the issue is to do with the spine or is] “bony”, it was very tender around the muscles where I felt him. I don't think we would do an MRI at the moment. From the examination that I did …

**P's Wife**: Yeah.

**Jnr Doc:** … the, erm, the neurological examination [that I conducted] was normal. I will talk to one of my seniors and see what they think.

**P's Wife:** I'd prefer it because mine was misdiagnosed.

**Jnr Doc:** Sure, I obviously will talk to one of my seniors.

**P's Wife**: Yeah.

**Jnr Doc:** But we won't just do an MRI unless it's warranted.

**P's Wife:** Well, normally, sort of pins and needles down the end is a sign that something is going on with the spinal cord.

**Jnr Doc**: Sure.

**P's Wife:** And, you know, as I say, because I've been through it myself, and I lost sensation in both my legs and arms.

**Jnr Doc:** Sure, OK.

**P's Wife:** It was nearly severed, so you can understand my concern.

**Jnr Doc:** Yeah. No, of course I can, and I will talk to one of my seniors, but we will only do it if they feel it's appropriate.

The doctor is not able to reassure the patient's wife about the suggested course of action and reiterates that she will speak with her ‘seniors’ (i.e., the consultant) and that the MRI would only be done if ‘it's appropriate’. In other cases across this study, companions also related their own ideas about what might be causing presenting symptoms and options for assessment or treatment based on their or the patient's previous healthcare experiences.

The doctor then takes the case to the consultant and explains the case presentation and history, outcomes of the physical examination and the wife's request:

**Jnr Doc:** His wife is extremely concerned that he needs an MRI. She had a disc prolapse and apparently, we misdiagnosed it and nearly paralysed her, so she wants him to have an MRI now. I've explained that we will only do that if we feel it is … appropriate … […]

**Consultant:** Fair enough.

**Jnr Doc:** So, I think … I mean, my initial … *[reading notes]* I was worried about the paraesthesia initially, erm, but I think it's reassuring that he's not got any other neurological …

**Consultant:** Any urinary symptoms?

**Jnr Doc**: No.

**Consultant**: Right.

**Jnr Doc:** Erm, so I thought maybe it was kind of a muscular spasm or possibly a slipped disc, but it's been going on for quite a long time, so …

**Consultant:** So, I guess my only concern is that this is … erm, if after he's had 10 mg of morphine for the pain […]

**Jnr Doc:** Yeah, and it's still not improving.

**Consultant:** … and he still can't lift his legs up off the bed because of that.

**Jnr Doc:** He can't.

**Consultant:** Then I think orthos [orthopaedic department] should see him. Especially his legs. […] they're the ones who are going to organise an MRI, if he needs an MRI. Erm. And at least then they can discuss with the wife as well about options.

**Jnr Doc:** Sure. OK, all right, thank you.

**Consultant:** Is that all right?

**Jnr Doc:** Yeah, that's good, thanks. So, I'm going to refer to orthopaedics *[Doctor and consultant nod and turn to walk away]*.

In relaying this case to the consultant, the doctor shares her considerations of what might be causing the patient's paralyzing pain, with the request for the MRI remaining central to the discussion throughout. The roles and practices of justifying resources and access to care pathways are demonstrated in this case.^5^ Together, the doctor and the consultant formulate a justification for not ordering an MRI while in ED care. This reflects the important role companions play in shaping interactions and decisions, both within the ED consultation room and beyond and across set care pathways.

### 3.2 Case 2: Older man presenting with breathlessness 

In this next case, an elderly man with breathlessness and a persistent cough has come to the ED with his wife, following a referral from his GP. The patient's wife participates in the consultation from the start when her husband defers to her to provide reasons for attending the ED as ‘she's better at explaining’. This happened in other cases in the study, where companions took part in the consultation by sharing the patient's case history, current medications and immediate and relevant events leading to arrival at the ED, prompting the patient to respond to the doctor and reassuring or confirming next steps for care.

Having established the primary concern and the nature and duration of various symptoms, the doctor begins outlining the purpose of myriad tests (X‐ray, echocardiogram [ECG], blood‐oxygen test) and setting expectations for the experience for the patient and his wife:

**Jnr Doc:** So, there's a couple of things that we need to do, some of them … right, first of all, we need a chest x‐ray, OK?

**Patient:** Right, yeah.

**Jnr Doc:** We'll get an ECG as well to see what your heart's doing, OK?

**Patient:** All right, yeah.

**Jnr Doc:** We do need to do … I know you've had blood tests done already, [**Patient:** yeah, ok] OK, but we need to do a different type of blood test. So that one comes from the vein, we need to do one from the artery *[**Patient** reacts, raises eyebrows, looks concerned]*, so we see what the oxygen levels are like in your body, essentially. *[**Patient** nods, half smiles]* Because you've been struggling to breathe so we need to see if your oxygen is getting around your body OK.

**Patient**: OK.

**Jnr Doc:** The problem with this test is we have to take it from the artery, normally from the one in your wrist. *[pointing at own wrist to show patient where the artery is]*.

**Patient:** Oh! *[grimaces].*

**Jnr Doc:** Yeah, so it hurts a bit more and we can't actually see the arteries, like the veins, so we have to feel and guess essentially!

**Ptnt's Wife**: Oh, dear!

**Jnr Doc:** So sometimes we don't get it straight away and sometimes it's a bit painful, but sometimes it's not so bad, so we'll just have to see.

**Patient**: Yeah.

**Jnr Doc:** OK? We'll do that test first and we'll get you off for a chest x‐ray and the ECG, you can get [that] done as well.

The patient's wife then asks:

**Ptnt's Wife:** So, you're not thinking of a blood clot?

**Jnr Doc:** *[Turning attention to wife]* So, it doesn't sound like a blood clot *[picks up notes]*, purely because you're *[speaking to patient]* saturating quite well on air.

**Ptnt's Wife**: OK.

**Jnr Doc:** But hopefully with the oxygen test *[pointing at wrist]*, if that oxygen level is OK, then that will kind of rule that out more than anything *[reading notes]*, but you can't rule it out.

The junior doctor continues to explain why the patient might be breathless, with a discussion of treatment for the immediate symptoms while waiting for the tests to support a diagnosis and facilitate ‘a good decision’ being made for further assessment, treatment, care and discharge from the ED.

**Jnr Doc:** *[Speaking to the patient]* So, there are lots of reasons why you could be short of breath, and you've got a bit of a wheeze on your lungs, so let's give you something to open up your lungs, just to help you with your breathing, OK? […] *[Speaking to patient and wife]* Then when we've got all the results back, then we'll have a good decision. *[**Patient** nodding]* OK? *[moves toward exiting the room, places notes on table]* So, let's get the blood tests done now, and then we'll get the x‐ray and the ECG done, yeah? *[leaving room]*.

**Ptnt's Wife:** Thank you very much.

Later, when the test results have come back, the patient has had an oxygen flow mask fitted and is sitting upright, where he had been lying further back before. The patient is subsequently admitted for overnight care, with the doctor first reporting the outcome the tests conducted. The doctor returns to the possibility that the patient's condition could be caused by a clot and reverses the assessment that the patient was doing well in terms of their level of oxygen saturation on air (as opposed to oxygen):

**Jnr Doc:** Essentially what we're looking at […] we were looking between those three diagnoses *[holding up three fingers and then listing on each]* essentially, like, whether it could be the infection that's carrying on, *[looking at wife]* a heart failing, or *[looking back at patient]* whether it's caused by a clot. Or whether it's caused by a clot.

**Patient:** A clot? *[Nod].*

**Jnr Doc:** A clot. So those are the three things that we're looking at. Looking at your x‐ray it looks more like an infection, OK? *[pointing at chest]* So, you can see the patchy lobes of infection *[gesturing]*, but it looks on both sides, so it's quite bad.

**Patient**: Is it?

**Jnr Doc:** Yeah, and that's probably causing you this drop in oxygen levels, OK? So, what we'll do is, we'll give you this [oxygen flow treatment] to help you open up your lungs a bit, and we'll give you some good medications [intravenous antibiotics] through there *[pointing to vein in arm]*, some good medications.

In both cases presented here and across the data set, companions and family members play important roles in offering information about patients' cases and engaging doctors in triadic consultations by being active participants.^23^, ^24^ Acknowledging and engaging with companions as integral members of the care interaction could enable doctors to communicate more effectively and collaboratively in the ED.

## 4 DISCUSSION

### 4.1 Communicating decisions to patients and their companions, and gatekeeping practices

In Case 1, the expectation that the patient's wife wants an MRI for her husband becomes a consideration for the junior doctor in taking the case forward.^11^, ^28^, ^29^ This demonstrates the important role companions play in shaping communication in ED consultations, and how this might be received by the doctor.^25^ The interjection from the patient's wife can be seen to divert the junior doctor from their goal‐oriented objective of efficiently moving the patient through ED pathways.^7^

The doctor moves the discussion back to the medical domain by explaining that following a ‘clinical’ examination, a decision will be made. The doctor also invokes the medical profession by stating that ‘we'll decide’, thereby positioning herself as part of a distributed network of decision‐makers and acting as a gatekeeper in being the primary point‐of‐contact for the patient.^9^, ^11^, ^18^, ^30^

In this case, the clinical reason for the referral to the orthopaedic department may well support an MRI being ordered, but this request is effectively being passed on to another department.^7^ Urgency for care and scarcity of resources mean clinicians justify, advocate and strategize for access to specific tests, decide to move patients into more appropriate specialist care (like orthopaedics) and communicate with patients and their families in ways that manage expectations.^5^, ^7^

Our findings reflect how doctors interacting with patients and companions in the ED continually collate and transform an array of information in reaching and communicating care decisions.^1^, ^2^ Companions can offer information about medical history, recent and related symptoms and advocate for care, for instance. They are active in the process and, in diverse and complex consultations, can play a crucial role in shaping communication and care decisions.^19^, ^21^ These decisions are enacted in a constrained environment where resource scarcity and considerations for managing capacity form part of healthcare organizational strategies and professional medical practice.^5^, ^12^ This is a discursive practice,^7^, ^18^ which extends into doctor–patient and companion interactions.

### 4.2 Medical knowledge, practice and justified care decisions

Case 2 is an illustration of the process of categorizing patients that defines ‘the ED as a service, as types of work and as particular kinds of patients’.^30^ The patient's wife's interjection of her ideas about the causes of her husband's illness (a clot) diverts the doctor from his goal‐oriented practice of collating information, explaining immediate next steps to be taken and resolving care,^7^ thereby breaching the ‘order’ of the clinic.

This interaction demonstrates a key role that patients' companions play in ED consultations and highlights the role of junior doctors as an interface between patients (and their companions) and broader organizational processes and systems of care that shape decisions taken in this setting.^11^, ^18^

In this instance, the complexity of care decisions and communication reflects a medical cosmology of external data in the form of test results and medical investigations being drawn into the narrative of the patient's case.^8^, ^12^, ^13^, ^18^ The visibility and value of specialized tests such as an X‐ray and what they can show (‘patchy lobes of infection’) or oxygen saturation tests also reflect this hierarchy in contradicting how well the patient appears.^8^ The patient's presence in the ED is justified by the route of treatment offered, particularly in being admitted for overnight care.^6^

We showed how decisions about care are constituted and legitimized and the construction of junior doctors' narratives of care decisions that are then communicated to patients and their companions.^8^, ^23^ In a hierarchy of medical knowledge, the use of tests and medical investigations outweigh the patient's (or companions) knowledge of their health or what is detected in an initial assessment or examination.

By researching communication in everyday care, we show that companions contribute to consultations. Companions can usefully provide case history, current or recent symptom onset, interpretation of care decisions and comfort for the patient,^23^, ^25^ which was reflected in this study. Sometimes, however, companions divert the expected, routinized goal‐oriented direction of doctor–patient consultations by querying decisions and advocating for care.^7^, ^8^, ^20^, ^31^, ^32^ Given their frequent presence in the ED, companions should be recognized as active participants in consultations as they are in other settings, such as primary and cancer care interactions.^23^, ^25^, ^32^, ^33^, ^34^ We contribute an understanding of doctors' responses to these interventions by family and companions and show how gatekeeping roles are enacted in communicating within and across set care pathways.^5^, ^28^, ^35^

## 5 CONCLUSION

This paper explores the complexity and dynamism of communication between junior doctors, patients and companions in a UK ED context. Through framework analysis of video‐ and audio‐recorded consultations, we explored the roles of junior doctors in moving patients through pathways in the ED. We showed how they convey care decisions in this context, and how patients and companions participate in this process.^21^, ^27^ The role of companions in consultations, and their effect on the consultation in shaping care interaction were additionally explored. Through the case studies, we showed how decisions are constituted, dispersed and communicated back to patients and their companions while informing them about their care.^7^, ^26^

In this setting, the junior doctor acts as the interface between the patient and their companions and various organizational, professional, medical and interpersonal factors that shape the decision of how the patient is moved along a care pathway and out of the ED.^5^, ^8^, ^13^, ^30^

### 5.1 Practice implications

The contribution of this paper has been to demonstrate the important role that family members and others who accompany patients play in shaping interactions and facilitating or redirecting the flow of ED consultations by querying, clarifying and requesting further information or care from doctors.^21^, ^23^, ^24^

Our findings reflect that doctors justify care decisions by communicating the next steps to the patient in a process to legitimize care offered in the ED that is characteristic of the clinical encounter.^14^, ^30^ Our findings confirm the recommendations of Cox and Li^19^ to embed communication with companions in clinical skills training and medical curriculum in ways that acknowledge the presence, participation and role of companions in ED medical consultations.

An area of further research could be to understand the role of companions in conveying information about the patient, their role in advocating for care and interpreting the decisions made regarding the patient's journey through the ED, and the implications for doctors' decision‐making and patient–clinician communication in this context.

## CONFLICTS OF INTEREST

The authors declare no conflicts of interest.

## Data Availability

Research data are not shared. The data are not publicly available due to privacy or ethical restrictions.

## 1

See Table A1.

**Table A1:** Coding framework for analysis—communicating care decisions in the emergency department.

Codes	Broad theme/topic
Demographic information	Case presentation
Patient	
Companion(s)	
Symptoms	
Steps prior to ED consult	
Duration of symptoms	
Case history	
Presenting condition	
Diagnosis	
Who is giving report (patient/companion)	
Treatment	Communicating decisions around care
Testing	
Referral to GP/community team	
Referral to other clinical department	
Admission for further treatment/assessment	
Explaining course of action	
Consultant/senior involvement	
	**Roles of doctors, consultants, patients, companions**
Directing discussion	Doctor
Speaking to patient	
Speaking to companion	
Speaking to consultant	
Speaking to other clinical team member	
Speaking to researcher	
Calling on clinical team/seniors/authority of broader team	
Responding to companion	
Collecting case presentation	
Sharing test results	
Sharing diagnosis	
Convey decision/outcome of consultation	
Telling case history	Patient
Telling experience leading up to arrival at ED	
Acknowledging tests/treatments/care decision being offered by doctor	
Asking for further care/explanation	
Telling case history	Companion
Telling experience leading up to arrival at ED	
Acknowledging tests/treatments/care decision being offered by doctor	
Asking for further care/explanation (advocating)	
Participating in care interaction	
Supporting decision of JD	Consultant
Suggesting justification for care offered	
Referral	
Symptom collation leading to diagnosis	Medical investigations/knowledge
Type of test	
Offered/not	
Reason given for offered/not	
Admitted to other dept for further investigation/care	
Discharge for community monitoring	
Tests showing more than symptom presentation	
Offering care/treatment/test on justified basis	Gatekeeping and justifying care decisions (resources)
Not offering care/treatment/test as not justified	
Reference to seniors/authority	
Testing guidelines	
Symptom presentation	
Pathway	
Referral to other depts for further assessment/access to testing/care	
**Companions present in interaction**	**Recommendations and suggestions for communication between patients and doctors in ED, with reference to companions**
Companions participating in interaction	
Companions offering information pertinent to care interaction	
Supporting/conveying decisions to patient alongside doctor	
Advocating for care	
Acknowledgement of complexity of communication in setting	
Roles of each (doctor, patient, companion, consultant) in moving through and across set care pathways in resource‐constrained environment	

## References

[1] Lawton R , Robinson O , Harrison R , Mason S , Conner M , Wilson B . Are more experienced clinicians better able to tolerate uncertainty and manage risks? A vignette study of doctors in three NHS emergency departments in England. BMJ Qual Saf. 2019;28(5):382‐388. 10.1136/bmjqs-2018-008390 PMC6560462 30728187

[2] Graham B , Endacott R , Smith JE , Latour JM . ‘They do not care how much you know until they know how much you care’: a qualitative meta‐synthesis of patient experience in the emergency department. Emerg Med J. 2019;36(6):355‐363. 31003992 10.1136/emermed-2018-208156

[3] Forero R , Nahidi S , de Costa J , et al. Perceptions and experiences of emergency department staff during the implementation of the four‐hour rule/national emergency access target policy in Australia: a qualitative social dynamic perspective. BMC Health Serv Res. 2019;19(1):82. 30700302 10.1186/s12913-019-3877-8 PMC6354365

[4] McKenna P , Heslin SM , Viccellio P , Mallon WK , Hernandez C , Morley EJ . Emergency department and hospital crowding: causes, consequences, and cures. Clin Exp Emerg Med. 2019;0(0):189‐195. 10.15441/ceem.18.022 PMC6774012 31295991

[5] Buchbinder M . Keeping out and getting in: reframing emergency department gatekeeping as structural competence. Sociol Health Illn. 2017;39(7):1166‐1179. 28422296 10.1111/1467-9566.12566 PMC5600633

[6] Willems DL . Balancing rationalities: gatekeeping in health care. J Med Ethics. 2001;27(1):25‐29. 11233373 10.1136/jme.27.1.25 PMC1733346

[7] Stevenson F , Pelletier C , Gibson W , Park S , Chrysikou V . The co‐construction of medical disposals in emergency medicine consultations. Soc Sci Med. 2018;218:69‐81. 30343178 10.1016/j.socscimed.2018.09.050

[8] Jewson ND . The disappearance of the sick‐man from medical cosmology, 1770‐1870. Int J Epedimiol. 1976;10(2):225‐244.

[9] Adams E , Goyder C , Heneghan C , Brand L , Ajjawi R . Clinical reasoning of junior doctors in emergency medicine: a grounded theory study. Emerg Med J. 2017;34(2):70‐75. 27340131 10.1136/emermed-2015-205650

[10] Fallowfield L , Jenkins V , Farewell V , Saul J , Duffy A , Eves R . Efficacy of a Cancer Research UK communication skills training model for oncologists: a randomised controlled trial. The Lancet. 2002;359(9307):650‐656. 10.1016/S0140-6736(02)07810-8 11879860

[11] Rapley T . Distributed decision making: the anatomy of decisions‐in‐action. Sociol Health Illn. 2008;30(3):429‐444. 18194358 10.1111/j.1467-9566.2007.01064.x

[12] Greaves D . Reflections on a new medical cosmology. J Med Ethics. 2002;28(2):81‐85. 11934934 10.1136/jme.28.2.81 PMC1733553

[13] Nettleton SJS . The emergence of e‐scaped medicine? Sociology. 2004;38(4):661‐679.

[14] Wamsiedel M . Reasonableness: legitimate reasons for illegitimate presentations at the ED. Sociol Health Illn. 2018;40(8):1347‐60. 29989187 10.1111/1467-9566.12776

[15] Vickers CH , Goble R , Lindfelt C . Narrative co‐construction in the medical consultation: how agency and control affect the diagnosis. Commun Med. 2012;9(2):159‐171. 24498700 10.1558/cam.v9i2.159 PMC3916960

[16] Street Jr., RL . How clinician–patient communication contributes to health improvement: modeling pathways from talk to outcome. Patient Educ Couns. 2013;92(3):286‐291. 23746769 10.1016/j.pec.2013.05.004

[17] Goodwin D . Decision‐making and accountability: differences of distribution. Sociol Health Illn. 2014;36(1):44‐59. 24112126 10.1111/1467-9566.12042

[18] Pelletier C , Chrysikou V , Gibson W , Park S , Stevenson F . The gift in A&E: re‐framing the medical case presentation through Mauss. Soc Theory Health. 2019;17:389‐406.

[19] Cox A , Li S . The medical consultation through the lenses of language and social interaction theory. Adv Health Sci Educ Theory Pract. 2020;25(1):241‐257. 30715620 10.1007/s10459-018-09873-2 PMC7018671

[20] Del Piccolo L , Goss C , Bottacini A , et al. Asking questions during breast cancer consultations: does being alone or being accompanied make a difference? European J Oncol Nurs. 2014;18(3):299‐304. 24629501 10.1016/j.ejon.2014.02.001

[21] Cordella M . A triangle that may work well: looking through the angles of a three‐way exchange in cancer medical encounters. Discourse Commun. 2011;5(4):337‐353.

[22] Laidsaar‐Powell R , Butow P , Bu S , Fisher A , Juraskova I . Attitudes and experiences of family involvement in cancer consultations: a qualitative exploration of patient and family member perspectives. Supp Care Cancer. 2016;24(10):4131‐4140. 10.1007/s00520-016-3237-8 27137213

[23] Laidsaar‐Powell RC , Butow PN , Bu S , et al. Physician–patient–companion communication and decision‐making: a systematic review of triadic medical consultations. Patient Educ Couns. 2013;91(1):3‐13. 23332193 10.1016/j.pec.2012.11.007

[24] Keeling DI , Laing A , De Ruyter K . Evolving roles and structures of triadic engagement in healthcare. J Serv Manag. 2018;29(3):352‐377.

[25] Pino M , Land V . How companions speak on patients’ behalf without undermining their autonomy: Findings from a conversation analytic study of palliative care consultations. Sociol Health Illn. 2022;44(2):395‐415. 35157323 10.1111/1467-9566.13427 PMC9306617

[26] Amelung D , Whitaker KL , Lennard D , et al. Influence of doctor‐patient conversations on behaviours of patients presenting to primary care with new or persistent symptoms: a video observation study. BMJ Qual Saf. 2020;29:198‐208. 10.1136/bmjqs-2019-009485 PMC7057803 31326946

[27] Gale NK , Heath G , Cameron E , Rashid S , Redwood SJ . Using the framework method for the analysis of qualitative data in multi‐disciplinary health research. BMC Med Res Methodol. 2013;13(1):1‐8. 24047204 10.1186/1471-2288-13-117 PMC3848812

[28] Nielsen SB . ‘And how long have you been sick?’: the discursive construction of symptom duration during acute general practice visits and its implications for ‘doctorability’. Time Soc. 2018;27(3):330‐349.

[29] Wolfe F , Clauw DJ , Fitzcharles M‐A , eds. 2016 Revisions to the 2010/2011 fibromyalgia diagnostic criteria. Seminars in Arthritis and Rheumatism. Elsevier; 2016. 10.1016/j.semarthrit.2016.08.012 27916278

[30] Hillman A . ‘Why must I wait?’ The performance of legitimacy in a hospital emergency department. Sociol Health Illn. 2014;36(4):485‐499. 24053721 10.1111/1467-9566.12072 PMC4579561

[31] Cox A , Rosenberg E , Thommeret‐Carrière A‐S , Huyghens L , Humblé P , Leanza Y . Using patient companions as interpreters in the Emergency Department: an interdisciplinary quantitative and qualitative assessment. Patient Educ Couns. 2019;102(8):1439‐1445. 30929764 10.1016/j.pec.2019.03.004

[32] Hanson J , Walthall K . Effects of hallway/corridor and companions on clinical encounters: a possible explanation. Emerg Med J. 2018;35:404‐405. 29886415 10.1136/emermed-2018-207467

[33] Miech EJ , Rattray NA , Flanagan ME , Damschroder L , Schmid AA , Damush TM . Inside help: an integrative review of champions in healthcare‐related implementation. SAGE Open Med. 2018;6:2050312118773261. 29796266 10.1177/2050312118773261 PMC5960847

[34] Turabian J , Rodriguez‐Almonte F , Minier‐Rodriguez L , Cucho‐Jove R , Villarin‐Castro A . Implications of companion presence with or without the patient in the family medicine consultation. J Fam Med. 2016;3(10):1093.

[35] Maynard DW , Heritage J . Conversation analysis, doctor–patient interaction and medical communication. Med Educ. 2005;39(4):428‐435. 15813766 10.1111/j.1365-2929.2005.02111.x

